# The experiences of the process of planning, starting and organizing a culturally specific nursing home for Finnish-speaking older persons: a qualitative study

**DOI:** 10.1186/s12913-020-05644-1

**Published:** 2020-08-19

**Authors:** Emina Hadziabdic, Katarina Hjelm

**Affiliations:** 1 Department of Health and Caring Sciences, Faculty of Health and Life Sciences, Linnaeus University, SE-351 95 Växjö, Sweden; 2 Department of Public Health and Caring Sciences, Uppsala University, SE-751 22 Uppsala, Sweden

**Keywords:** Culturally specific nursing home, Cultural diversity, Elderly migrants, Qualitative study, Transcultural nursing

## Abstract

**Background:**

Globally there are growing multicultural and multilingual societies. As a result of extensive international migration, the number of elderly migrants has increased and will further increase in the future. This makes it necessary for elderly healthcare services to meet elderly migrants’ healthcare needs concerning language and cultural barriers. To our knowledge, previous research in the area of culturally specific nursing homes for migrant seniors is still limited. Thus, the study aimed to investigate the experiences of planning, starting and organizing a culturally specific nursing home for Finnish-speaking older persons.

**Methods:**

An explorative qualitative study using both semi-structured individual interviews and focus group interviews as data collection. Thirteen informants were purposively recruited, two from Finnish-speaking association, seven healthcare professionals and two family members. Data were analysed by qualitative content analysis.

**Results:**

Three categories, each with sub-categories, emerged from the data: 1) Motivation to develop this particular culture-specific nursing home; 2) Organizational issues and 3) Aspirations for the future. The study found that information from policy makers, the localization and activities of the nursing home, having healthcare staff who speak the minority language, organizing the nursing home as a mixture of older members of both the majority and the minority communities, all affected the planning, starting and organization of a culturally specific nursing home.

**Conclusion:**

This study found that information, localization, activities and language adapted to elderly migrants affected the planning, starting and organization of a culturally specific nursing home for Finnish-speaking older persons. These findings should support the healthcare organization in planning, managing and organizing sustainable nursing home care for older people belonging to a minority in order to attain the aim of person-centered and equal healthcare.

## Background

The ageing population, over 65 years, is increasing rapidly in developed nations, as is international migration [1, 2]; as the result, 19% of the population of Sweden are migrants [3]. Taken together, these two developments make increasing demands on healthcare. Migrants in Sweden are a very heterogeneous group, currently comprising people who came to Sweden as labour migrants when they were young and have aged in Sweden (mainly from Finland), as well as those persons who have migrated in older age as refugees (migrants mainly from former Yugoslavia, the Middle East, Afghanistan, North Africa and Syria). The largest group of migrants over the age of 65 are from Finland, approximately 30% [3]. The heterogeneity of the older population highlights the requirement to offer a range of services, e.g. nursing homes that consider elderly persons’ language, cultural and religious differences [4]. In Sweden, there are five national minorities: Jews, Roma, Sami, Swedish Finns and Tornedalers [5]. Common to the minority groups is that they have lived in Sweden for a long time and that they constitute groups with an obvious kinship. In an administrative area minority groups have special rights such as the entitlement to receive all or part of the care from staff who speak the minority language, and they also have the right to use a minority language in contact with authorities [6]. This law, along with others laws such as the Social Services Act [7], the Health and Medical Services Act [8] and the Patients Act [9], emphasizes that healthcare services for older persons should be adapted to the needs of older people. The authorities in the administrative area are obliged by law to ensure that there are healthcare staff in the administrative areas that speak the minority languages. The administrative areas firmly assert the individual’s right to care in a minority language, but they have practical difficulties when it comes to offering elder care in minority languages because lack of staff skilled in these languages [5]. Culturally specific nursing homes for the elderly focus on issues such as the acknowledgement of specific food, holidays, customs and language, and organizational issues such as workforce and the environment.

It is recognized that key factors helping residents to accept and adjust to the elderly care home include creating a sense of home, maintaining self-identity and self-worth and developing a positive relationship with staff [10]. A previous literature review [11] focusing on culturally specific and mainstream long-term care facilities for migrant seniors found that older migrants have identified the wish to remain connected with their cultural routines and identity as important factors. Another study [12] investigated the best practice of providing person-centred care for residents with moderate to advanced dementia from diverse ethnic backgrounds and showed that it was associated with residents’ perceptions of their individual preferences and feelings of belonging. Further, other previous research focusing on residents’ and family members’ perceptions of cultural diversity in aged care homes [13] found that they were generally satisfied with the cultural and linguistic diversity in care homes.

Previous studies showed that native language, a shared ethnic background with the staff and shared customs increase the older people’s well-being [14–17]. Iranian relatives stated that a profiled nursing home was an adequate option but on the other hand they often felt a push-pull conflict between emotion and reason [18]. Further, collaboration between representatives of the municipality, Finnish-speaking migrants’ associations and staff at the nursing home influenced the development of a culturally specific nursing home for older Finnish-speaking people [17]. Although we have found studies concerning culturally specific nursing homes [14–18], no study has been conducted on how family members, healthcare professions and representatives from migrants’ association experience planning, starting and organizing a culturally specific nursing home for Finnish-speaking older persons. Thus, there is still a gap in research concerning planning, starting and organizing of nursing home services and resources to meet individual preferences among older migrants/minorities. This issue is important to investigate in order to support aging in the right place, establishing the human rights of care-dependent older migrants, enchaining older migrants’ dignity and their ability to make choices [2].

This study focused on a culturally specific nursing home that belongs to a Finnish administrative area in a municipality in Sweden and studied the experiences of planning, starting and organizing a culturally specific nursing home for Finnish-speaking older persons.

### Aim

The aim of the study was to investigate the experiences of planning, starting and organizing a culturally specific nursing home for Finnish-speaking older persons. Research question for the study was:

1. How did healthcare professionals, the community and family members experienced planning, starting and organizing a culturally specific nursing home for Finnish-speaking older persons?

## Methods

### Design

This study is an explorative qualitative study using both semi-structured individual interviews and focus group interviews to investigate the experiences of establishing a culturally specific nursing home from different perspectives: those of healthcare professionals, the community and family members to get a variety of experiences and to arrive at a better understanding of the studied phenomenon [19].

### Setting

The studied nursing home was placed in a municipality that belonged to a Finnish administrative. The culturally specific nursing home was started in 2013 and located close to nature and transport facilities and was organized as a mixture of Swedish and Finnish-speaking older persons. Finnish activities such as singing, baking and cooking are represented at the nursing home and provides with help of Finnish-speaking pensioners and Finnish associations. Further, the Finnish-speaking healthcare staff such as a registered nurse and two assistant nurses were employed at the nursing home (for more details see Hadziabdic and Hjelm [17]).

#### Procedure and participants

A purposive sample was chosen to ensure variation in age, gender, language, and different healthcare professional backgrounds and to get deep and rich information and better understanding about the phenomenon of interest [19].

The project coordinator and the project manager for the Finnish administrative area in the municipality were contacted by telephone by the author, EH, to inform about the study and to obtain permission to implement it. After approval, they were requested to invite representatives from Finnish-speaking associations who had experiences of establishing a culturally adapted nursing home to participate in the study. Further, the manager of the nursing home was contacted by telephone by the author, EH, to inform about the study and to obtain permission to implement it. After approval, the manager was requested to invite different healthcare staff at the nursing home and family members of the Finnish-speaking residents to participate in the study. The project manager and project coordinator of the Finnish administrative area, and the manager of the culturally specific nursing home mediated contact information for those interested in participating to the author EH, who contacted them to set a time and place for the interview.

The study included 13 persons, two from the Finnish-speaking association, three Finnish-speaking assistant nurses, three Swedish-speaking assistant nurses, a Finnish-speaking registered nurse, a Swedish-speaking registered nurse, two family members and a manager at the culturally specific nursing home (see Table 1).

**Table 1:** Characteristics of the study population

Variable		Persons (*N* = 13)
Gender *(n)*	Female	13
Age (years) ^a^	36–45 years	1
	46–55 years	7
	56–69 years	5
Work experience in current nursing home (years)^a^		4 (0,5 years–23 years)
	0.5–5 years	5
	6–10 years	1
	11–15 years	0
	16–20 years	1
	21–25 years	2
Professional level	Registered nurse	3
	Assistant nurse	7
	Registrar	1
	Missing information	2
Position in hierarchy	Employee	10
	Manager	1
	Pensioner	2
Place of birth	Finland	5
	Sweden	7
	Syria	1

^a^Values are median (range)

#### Data collection

Data were collected using both individual interviews and focus group interviews with an interview guide developed for this study and based on findings from a previous study [17]. The interview guide focused on experiences of planning, starting and organizing the culturally specific home for Finnish-speaking persons. Examples of the main questions were: “Please, describe the way of planning for this culturally specific nursing home for Finnish-speaking older persons?”, “Please, describe the way of starting for this culturally specific nursing home for Finnish-speaking older persons?” and “Please, describe the way of organizing for this culturally specific nursing home for Finnish-speaking older persons?” The follow questions was: “who started/planned/organizing it?, “Why?” How did you and others react?,” How did you feel?”. Using different qualitative methods of data collection such as individual and focus group interviews made it possible to generate data of depth and by interaction in the group to reveal a participant’s more or less unconscious experiences and opinions [19, 20]. Further, using individual interviews prevented the anxieties related to power relation in groups, which might lead to limited opportunities for some informants to talk about their experiences in front of group or manager.

The individual interviews (*n* = 5) were held by telephone and lasted approximately 30 min. Further, three focus group interviews were conducted at the culturally specific nursing home in secluded venues chosen by the informants. One group included two participants and two groups included three participants (*n* = 8). In order to promote a relaxed and dynamic interviews climate the researcher chose the interview format based on practical reasons (e.g. travel distance), possibility to reach the aim of the study and to avoid negative influence of power relations in the groups.

Participants in the focus groups were planned to be fairly homogeneous in terms of profession, gender and language [20]. Thus, the focus group interaction was active and the interviews lasted about 60 min. Both individual and focus group interviews were held by the first author, a nurse and researcher in nursing science with experience of migrants’ health and healthcare issues.

All interviews were audiotaped and transcribed verbatim by a professional secretary and then analysed.

#### Data analysis

The data used qualitative inductive content analyses to analyse the data in order to identify patterns and to discover relationships between experiences [19] with the aid of the qualitative analysis software Atlas Ti (ATLAS.ti Scientific Software Development GmbH).

The transcripts from the interviews were read through thoroughly several times to achieve a sense of the whole [19]. The texts were then broken into smaller meaning units. The next step was to search for similarities and patterns to develop sub-categories and categories from the context of the meaning units. The first author investigated the texts for regularities, contradictions, similarities and patterns, returning to the data analysis and rereading all the transcripts until no new information was found. Categories were developed, modified and refined until an acceptable system of coding data was recognized. In order to ensure credibility, the first author analysed the data, and the second author reviewed and checked the content and grouping of the categories and dimensions. In naming categories, concepts as close as possible to the text were used in order to ensure confirmability. Analysis of data proceeded until no new information was acquired (see Table 2) [19].

**Table 2:** Example over the number of codes mentioned in each category and sub- category

Categories	No. of codes	Sub-categories	No. of codes
Motivation to development of the current culture-specific nursing home	18	Initiative, information and access	12
		Localization and language preferences	6
Organizational issues	87	Adjustment to provide culturally specific services based on Finnish-speaking cultural background	26
		Organization and activities at the nursing home	29
		Work environment for the staff	12
		Partnership between staff at the nursing home, Finnish-speaking migrant associations, representatives of the municipality and family members	20
Aspirations for the future	20	Adjusting to culturally adapted service at the current nursing home	19
		Independent administrative area	1
**Total number of codes**	**125**		

## Results

Three categories, each with sub-categories, emerged from the data:

1) Motivation to development of the current culture-specific nursing home; 2) Organizational issues and 3) Aspirations for the future. The categories and sub-categories are supported by illustrative quotations.

### Motivation to development of the current culture-specific nursing home

#### Initiative, information and access

Some participants stated that the initiative to start the culture-specific nursing home for Finnish-speaking older persons came from the Swedish-Finnish-speaking community in the municipality. The reason was the many years’ experience of being migrants in Sweden which included better knowledge of laws and rights in Sweden. However, the initiative for the culture-specific nursing home was unknown to most of the participants.

*"I think that it is the Finnish association here in town that has been pushing and complaining to the municipality that they want a wholly Finnish old people’s home … that we came here after the fifties, sixties and did the most difficult jobs and … you get all the dirty jobs … then of course you can stand up for yourself and you have learned how to live in the country and what rights and opportunities you have … ”.*

Some participants described how information provided during the establishment of the nursing home for Finnish-speaking older people stated that the home would be organized as a culturally specific service exclusively for Finnish-speaking persons. In reality, the home came to be organized with a mixture of Swedes and Finnish-speaking persons. Some staff said that the incorrect information meant that the staff needed to handle family members’ dissatisfaction with the home not being organized for Finnish-speaking persons only.

To become a resident at the home, the case has to be processed according to set routines followed by the municipality. There is a queue system which means implies that the person with the greatest need may become a resident of the home and there is no guarantee that this will be a Finnish-speaking person.

*“It is the need that governs … . There is a waiting list, and it is the one who has the greatest need who gets in and it is not certain that it will be a Finnish speaker”.*

#### Localization and language preferences

The wish to become a resident of the culturally specific nursing home also depends on the location of the home. The home is in an area where many Finnish-speaking persons live. Further, the fact that Finnish-speaking staff are available at the home is another aspect motivating the choice of this home for the older persons.

*“ … .And he* (dad) *has the opportunity to talk Finnish with others … that he has all this Finnish and he seems happy to be there too, so that is very good. Yes, I think that he can’t speak Swedish anymore … he has pretty much forgotten about … that he can, like, converse with someone in Finnish it … it is worth its weight in gold”*

### Organizational issues

#### Adjustment to provide culturally specific services based on Finnish-speaking cultural background

Informants expressed that the home’s organization as mixture of Swedish and Finnish-speaking persons and the availability of Finnish-speaking staff, customized diet and cultural activities were positive aspects of the home’s organization in order to be able to preserve customs and habits, and to make themselves understood, which in turn prevents isolation. Further, they said that the need to have Finnish-speaking staff available was dependent on the older people’s knowledge of Swedish and on their specific illness. Some informants said that older people who suffer from dementia need to have someone they recognize, in this case Swedish-speaking staff, as it can be more difficult with unknown staff even if they speak Finnish with them.

*“ … it also depends on diseases. We had Finnish-speaking staff but it was not possible so I* (not Finnish-speaking) *had to go in and help him because he recognized me. He was more confident with me than with her, though she spoke Finnish with him … .”*

#### Organization and activities at the nursing home

Informants stated that the culturally specific nursing home was organized as a mixture of Swedish and Finnish-speaking residents in the department. Some of the Finnish-speaking informants did not like this organization; they felt that Finnish-speaking residents should be located in one department where they could speak Finnish and watch Finnish television in public rooms. Participants mentioned that some of the Swedish residents were disappointed when they spoke Finnish in public rooms at the department.

*“other residents at the home may be a little annoyed when they speak Finnish at the dining table, for example.”*

The home was staffed with a Finnish-speaking registered nurse and three assistant nurses, with a fixed schedule and placement. The planning of the time schedules was done so that one Finnish-speaking staff member should always be present at the nursing home to assist with language barriers. However, it was not always possible to fulfil the requirement to have a Finnish-speaking staff member present at the home due to difficulty in recruiting them.

*“That was the aim* (to have Finnish-speaking staff at the home) *but sometimes it is not possible to arrange, it fails because there is a huge shortage of staff … . staff who speak Finnish … … It is not, but it is deficient … eh we say in dad’s staff … I don’t think there is anyone who speaks Finnish … ”*

Informants described how activities related to Finnish culture, such as singing, baking and cooking to mark holidays, are organized by the Finnish-speaking association in cooperation with Finnish-speaking staff, while staff who do not speak Finnish were not involved.

#### Work environment for the staff

Informants said that it was no different working at the culturally specific home in comparison to other elderly homes which were not adapted to a specific culture. Finnish-speaking staff found it positive to use their native language and their knowledge of Finnish history in caring. In contrast, non-Finnish-speaking staff found it negative when they could not communicate with Finnish-speaking residents.

*“I don’t see any difference in the care they need, they get it. Everyone has different needs as regards what kind of care they need. Everyone gets what they need, be it Swedish or Finnish or other nationalities.*

*I agree.*

*They live here because they have needs “ (Focus group interview, Swedish-speaking assistant nurses )*

*“I think it is very good … . it is a way for me to keep the language. … then I have to use the language in my job.”*

*“There are times* (difficulties) *when you can’t understand each other. It’s very frustrating.”*

#### Partnership between staff at the nursing home, Finnish-speaking migrant associations, representatives of the municipality and family members

Partnership between the parties involved was described as mostly good. Representatives of the Finnish-speaking association were disappointed with the cooperation with representatives from the Finnish-speaking administrative area as they felt that they were not involved when discussing plans for the culturally specific home. Instead, they received information about only what was done.

*“ … No … she* (person from the Finnish administration at the municipality) *… she says what she has done but she does not say … what she is planning or what to do, but then when it’s done she says here it is … ”*

### Aspirations for the future

#### Adjusting to culturally adapted service at the current nursing home

Some informants said that they would like the nursing home to be organized in the future so that all older Finnish-speaking persons should be in the same department of the nursing home, with Finnish-speaking staff available around the clock, with customized diet and cultural activities every day. Some informants felt that the highest wish was to develop the culturally profiled nursing home solely for Finnish-speaking persons. In contrast, some informants wished that all nursing homes were culturally adapted and not just some organized as nursing homes with a specific cultural profile.

*“Not to divide people as if there were something special about being Finnish, or there is something special about being Swedish.*

*All are of equal worth …*

*… In such cases I think of a multicultural home with all nationalities so that there would be no groupings.”*

#### Independent administrative area

Informants from the Finnish-speaking association wished that the administrative area should be an independent institution and not belonging to a municipality in order to have better and impartial partnership.

*“ … what the old … the old … what is it called … the trade union meetings that … the representative was very much on the workers’ side but then when they came to the (laughter) managers …*

*… the managers were on their side.*

*… we feel the same … we all feel the same way … that she* (person from the Finnish administration at the municipality) *sides more with the municipality so she wants to sit there and make decisions with the municipality.*

*… so she is not wholeheartedly on our side … ”*

## Discussion

Our findings, indicated that factors such as information from policy makers, nursing home localization and activities, healthcare staff who speak the minority language, the organization of the nursing home as a mixture of older members of both the majority and the minority communities, affected the planning, starting and organizing of a culturally specific nursing home to meet individual preferences among older migrants. As the literature review did not expose any earlier studies on this, only partial comparisons to other studies will be possible.

The findings highlights the influence of communication and information from policy makers to persons in the minority communities especially to avoid minority persons’ dissatisfaction with the home being organized as a mixture of Swedish and Finnish-speaking residents in the department and not as culture-specific service for Finnish-speaking older persons only. The reason for this organization was that only a limited number of older Finnish-speaking persons registered an interest in being a resident in the Finnish culturally specific nursing home in the establishment process, so it was decided to organize the home with a mixture of Swedes and Finns [17]. This study also showed that language and cultural barriers were not the only factors [21] explaining the miscommunication. The described irritation evolved due to a gap between the concept (Finnish-speaking only) and the actual implementation (opening up to Swedish-speaking residents), not because the families had difficulties in understanding the information provided. Finnish-speaking participants in this study spoke fluent Swedish and have lived in Sweden the greater part of their lives. Minority elderly persons have been shown to have less knowledge about how to access to elderly care services [21] and they are mostly persons with low health literacy who lack knowledge and skills about the healthcare system [22]. Therefore information provided about organization of culturally specific nursing homes should be adapted to minority elderly as well as their networks levels of health literacy [21], for example by providing information in different formats such as oral, written and in pictures [23] in order to avoid healthcare gaps [24].

Another important finding of this study is the effect of the location of the nursing home, multicultural and multilingual staff within the nursing home organization and cultural activities available at the nursing home. These factors seem to play an important role in the planning, starting and organization of the nursing home for older Finnish-speaking people in a Finnish administrative area in Sweden. Overcoming language barriers among multilingual staff within the nursing home care organization [17, 25] in familiar socio-cultural circumstances increases the well-being of minority people [14, 15] and their relatives [18]. This new finding showed that local policy makers should be aware of factors such as location of the nursing home, and how language and communication and cultural beliefs and customs influence the organization of appropriate care for older minority persons. Further, they should take into account people’s specific needs based on cultural background in order to provide appropriate and high-quality care [26].

### Limitations of the study

One potential limitation of the study was the size of the focus group, from 2 to four, and could be seen as a weakness in terms of the results. However, the reason to interview in two people in a group, Finnish-speaking pensioners who are engaged in Finnish culturally activities at the nursing home, with particular knowledge of the experiences of planning, starting and organizing process was to get additional in depth-and rich information about the particular aim. It has been identified that smaller groups is easier to host, are more relaxed for participants [20] and the most important is not the group size but the interaction in the group [27] which was lively.

Another potential limitation of the study was that the study did not include perspectives from the Finnish-speaking older people living in the nursing home. However, it was not possible as the Finnish-speaking older people in the home at the time of the investigation suffered from dementia: this needs to be further study.

## Conclusion

This study has addressed several issues related to planning, starting and organizing a culturally specific nursing home for Finnish-speaking older persons. The results can help the healthcare organization to facilitate sustainable nursing home care for minority older people including the information provision strategies adapted to the health literacy of minority older persons and their family members, overcoming communication barriers by ensuring multilingual staff at the nursing home and providing appropriate care in familiar socio-cultural circumstances in collaboration with minority communities. Further, findings from this study are contextual and cannot be generalized as a result of the qualitative approach but as statements from several healthcare professionals, the community and family members gave a similar picture, the findings gave a deeper understanding and can be transferred to other minorities or multicultural /−lingual groups than Finnish-speaking with similar characteristics.

## Data Availability

In order to protect the integrity, anonymity and confidentiality of the respondents, data will not be shared.

## References

[1] Spencer S, Martin S, Bourgeault YL, O’Shea E (2010). RS No. 41 - The Role of Migrant Care Workers in Ageing Societies Report on Research Findings in the United Kingdom, Ireland, Canada and the United States.

[2] WHO (2015). WHO: World Report on Ageing And Health. In*.* Luxemburg.

[3] Utrikes födda i Sverige [https://www.scb.se/hitta-statistik/sverige-i-siffror/manniskorna-i-sverige/utrikes-fodda/].

[4] National Board of Health and Healthcare (2015). Äldre omsorg på minoritetsspråk (Elderly care in minority languages) In*.*: National Board of Health and Healthcare.

[5] Länstyrelsen i Stockholms län (2017). Nationella minoriteter- Minoritetspolitikens utveckling år 2016.

[6] Swedish Parliament (2009). Lag om rätt om nationella minoriteter och minoritetsspråk (Act on national minorities and minority languages). In*.* Edited by culture Mo.

[7] Swedish Parliament (2001). Socialtjänstlag (Social Service Act).

[8] SFS (2017). Hälso- och sjukvårdslag (Health and Medical Services Act).

[9] SFS (2014). Patientlag (Patient Act). Sveriges riksdag (The Swedish Parliament).

[10] Falk H, Wijk H, Persson LO, Falk K (2013). A sense of home in residential care. Scand J Caring Sci.

[11] Montayre J, Montayre J, Thaggard S (2018). Culturally and linguistically diverse older adults and mainstream long-term care facilities: integrative review of views and experiences. Res Gerontol Nurs.

[12] du Toit SHJ, Buchanan H (2018). Embracing Cultural Diversity: Meaningful Engagement for Older Adults With Advanced Dementia in a Residential Care Setting. Am J Occup Ther.

[13] Xiao LD, Willis E, Harrington A, Gillham D, De Bellis A, Morey W, Jeffers L (2017). Resident and family member perceptions of cultural diversity in aged care homes. Nurs Health Sci.

[14] Heikkila K, Ekman SL (2003). Elderly care for ethnic minorities--wishes and expectations among elderly Finns in Sweden. Ethn Health.

[15] Andrews RA (2012). Anglo-Indian residential care homes: accounts from Kolkata and Melbourne. J Cross Cult Gerontol.

[16] Heikkila K, Sarvimaki A, Ekman SL (2007). Culturally congruent care for older people: Finnish care in Sweden. Scand J Caring Sci.

[17] Hadziabdic E, Hjelm K (2018). Establishing a culturally specific nursing home for Finnish-speaking older persons in Sweden: a case study. Nurs Open.

[18] Kiwi M. Embracing Cultural Diversity: Meaningful Engagement for Older Adults With Advanced Dementia in a Residential Care Setting. Dementia (London). 2017;1471301217743835. 10.5014/ajot.2018.027292 30760401

[19] Patton MQ (2015). Qualitative research & evaluation methods : integrating theory and practice 4.Th ed. edn.

[20] Krueger RA, Casey MA (2015). Focus groups : a practical guide for applied research, 5th edition. Edn.

[21] Suurmond J, Rosenmoller DL, el Mesbahi H, Lamkaddem M, Essink-Bot ML (2016). Barriers in access to home care services among ethnic minority and Dutch elderly - a qualitative study. Int J Nurs Stud.

[22] Kutner M, Greenburg E, Jin Y, Paulson C (2006). The Health Literacy of America's Adults: Results from the 2003 National Assesment of Adult Literacy. Institute od Education Sciences, National Center for Education Statistics.

[23] Warburton J, Bartlett H, Rao V (2009). Ageing and cultural diversity: policy and practice issues. Aust Soc Work.

[24] Misra-Hebert AD, Isaacson JH (2012). Overcoming health care disparities via better cross-cultural communication and health literacy. Cleve Clin J Med.

[25] Hadziabdic E, Lundin C, Hjelm K (2015). Boundaries and conditions of interpretation in multilingual and multicultural elderly healthcare. BMC Health Serv Res.

[26] World Health Organization., United Nations. Office of the High Commissioner for Human Rights (2008). Human rights, health, and poverty reduction strategies.

[27] Tang KC, Davis A (1995). Critical factors in the determination of focus group size. Fam Pract.

[28] Förordning om etikprövning av forskning som avser människor (Swedish law: Regulation of ethical research involving humans) [http://www.riksdagen.se/webbnav/index.aspx?nid=3911&bet=2003:460 (accessed 15 Mar 2010).].

[29] Declaration of Helsiniki- Ethical Principles for Medical Research Involving Human Subjects [https://www.wma.net/policies-post/wma-declaration-of-helsinki-ethical-principles-for-medical-research-involving-human-subjects/].

